# Identification and Characterization of Metabolic Subtypes of Endometrial Cancer Using a Systems-Level Approach

**DOI:** 10.3390/metabo13030409

**Published:** 2023-03-09

**Authors:** Akansha Srivastava, Palakkad Krishnanunni Vinod

**Affiliations:** Centre for Computational Natural Sciences and Bioinformatics, IIIT, Hyderabad 500032, India

**Keywords:** metabolic reprogramming, endometrial cancer, transcriptome, systems biology, reporter metabolites

## Abstract

Endometrial cancer (EC) is the most common gynecological cancer worldwide. Understanding metabolic adaptation and its heterogeneity in tumor tissues may provide new insights and help in cancer diagnosis, prognosis, and treatment. In this study, we investigated metabolic alterations of EC to understand the variations in metabolism within tumor samples. Integration of transcriptomics data of EC (RNA-Seq) and the human genome-scale metabolic network was performed to identify the metabolic subtypes of EC and uncover the underlying dysregulated metabolic pathways and reporter metabolites in each subtype. The relationship between metabolic subtypes and clinical variables was explored. Further, we correlated the metabolic changes occurring at the transcriptome level with the genomic alterations. Based on metabolic profile, EC patients were stratified into two subtypes (metabolic subtype-1 and subtype-2) that significantly correlated to patient survival, tumor stages, mutation, and copy number variations. We observed the co-activation of the pentose phosphate pathway, one-carbon metabolism, and genes involved in controlling estrogen levels in metabolic subtype-2, which is linked to poor survival. *PNMT* and *ERBB2* are also upregulated in metabolic subtype-2 samples and present on the same chromosome locus 17q12, which is amplified. *PTEN* and *TP53* mutations show mutually exclusive behavior between subtypes and display a difference in survival. This work identifies metabolic subtypes with distinct characteristics at the transcriptome and genome levels, highlighting the metabolic heterogeneity within EC.

## 1. Introduction

Endometrial cancer (EC) is the most common gynecological cancer worldwide. EC emerges in the inner lining of the uterus and may spread to the outside of the uterus in advanced stages [1]. The number of EC cases is increasing, with major risk factors being infertility, obesity, and sedentary lifestyles [2]. Traditionally, EC is classified into Type 1 and Type 2 based on clinical, endocrine, and epidemiological observations, or into endometrioid, serous, and clear-cell adenocarcinomas based on histological characteristics [3]. Type 1 ECs are mostly endometrioid adenocarcinomas, estrogen-dependent, and have better survival outcomes [4]. In contrast, Type 2 ECs are predominately serous carcinomas, estrogen-independent, and have a poor survival rate [4]. However, the traditional classification system may not fully capture the complexity of cancers and are prone to differential diagnosis due to interobserver variation between pathologists [5,6]. A comprehensive genomic characterization of EC by the Cancer Genome Atlas (TCGA) provides opportunities for molecular classification of EC [7]. An improved understanding of how various molecular alterations contribute to EC patients’ survival will assist with treatment and therapeutic development. In this work, we hypothesized that EC patients could be stratified into metabolic subtypes based on gene expression profiles to predict patient survival.

Metabolic reprogramming is one of the emerging hallmarks of cancer [8]. Cancer cells rewire their metabolism to fulfill their increased demand for nutrients and energy. Cancer cell metabolism is affected by different intrinsic factors, such as mutations, epigenetics, cellular composition, and microbial populations [9]. It is also influenced by extrinsic factors, such as tumor microenvironment, obesity, and diet [9]. Metabolic phenotypes transform over time as tumor growth progresses from early to late stages [9]. These factors contribute to metabolic heterogeneity, resulting in the diverse metabolic profiles of cancer patients. Therefore, comprehending the variations in metabolic processes is crucial.

Different pan-cancer studies have used transcriptomic data of adjacent normal and tumor samples to study metabolic pathway changes across various cancers [10,11,12,13,14]. EC samples have significantly upregulated genes involved in carbohydrate metabolism, serine biosynthesis, fatty acid metabolism, TCA cycle, and glutaminolysis. Studies focusing on EC metabolism used differentially expressed genes between adjacent normal and tumor samples to build nomograms for the prognosis of cancer patients [15,16]. Alterations in choline homeostasis have been reported in EC using the nuclear magnetic resonance (NMR) technique [17]. These studies are limited to metabolic alterations between adjacent normal and tumor samples. The metabolic heterogeneity of EC patients is yet to be explored, and it requires an unsupervised approach without prior class label information to identify metabolic subtypes. A systems-level characterization of metabolic subtypes will provide a mechanistic understanding of the disease.

Genome-scale metabolic models (GEMs) have been widely used for studying metabolism at the system levels with applications in biotechnology and medicine [18,19]. A GEM is a metabolic network comprising of enzymes, reactions, and metabolites, representing all biochemical reactions of an organism’s metabolism under given conditions. It serves as a scaffold for the integration of omics data. The constraint-based modeling approach (flux balance analysis) allows for integrating omics data with GEMs to generate disease-specific models [20,21]. The metabolic network topology from GEM reconstruction can also be used for the integration of omics data to identify metabolic hotspots and subnetworks specific to a disease condition [22,23,24].

In this work, we analyzed the transcriptomic data of EC patients to identify the metabolic subtypes of EC. A generic human genome-scale metabolic model was used as a scaffold for subtype identification and characterization. We also examined the characteristics of each subtype at the genomic level. Our study provides subtype-specific biomarkers and reporter metabolites, which will be helpful in the diagnosis and prognosis of EC.

## 2. Materials and Methods

### 2.1. Datasets and Data Pre-Processing

We downloaded the TCGA RNA-Seq data (HTSeq Count) of EC using TCGAbiolink [25]. The TCGA MC3 files were retrieved from The Genomic Data Commons (GDC) portal to analyze the mutation profile of EC patients. The cBioportal was used to obtain the segmented copy number variation datasets [26]. We considered TCGA-Clinical Data Resource (CDR) to retrieve the corresponding clinical annotation for EC patients [27]. For validation, the microarray series dataset (GSE17025, Affymetrix Human Genome U133 Plus 2.0 Array), consisting of 91 EC samples, was downloaded from the NCBI GEO database using the GEOquery package [28]. We employed the human genome-scale metabolic model (HMR2.0) to study cancer metabolism. The model consists of 8181 reactions, 6006 metabolites, and 3765 genes, which describe the standard metabolic processes of a human cell.

The TCGA RNA-Seq dataset consists of 565 samples, of which 542 are primary tumor samples (sample type code—01) and 23 are tumor-matched normal samples (sample type code—11). We used variance-stabilizing transformation (VST) to normalize the RNA-Seq raw count data. The validation microarray dataset comprises 79 endometrioid and 12 papillary serous samples with various grades. We normalized the microarray data using Affymetrix’s MAS5.0 algorithm and log_2_ transformation.

### 2.2. Identification of Metabolic Subtypes

**Figure 1:**
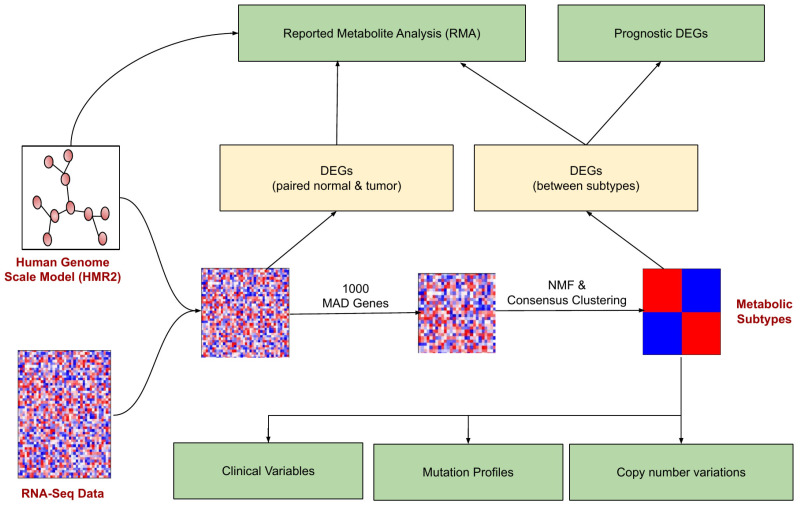
The workflow for metabolic EC subtype identification and characterization. The rectangular boxes (green) represent the study’s main outcomes: understanding the differences between EC metabolic subtypes (at clinical, mutation profile, and copy number variations) and identifying the reporter metabolites and prognostic DEGs.

The pipeline for identifying and characterizing metabolic subtypes of EC using the transcriptomic data is shown in Figure 1. The metabolic genes present in the human genome-scale metabolic model (HMR2.0) were considered for the analysis. HMR2.0 has been extensively used for generating metabolic models of normal tissues and disease conditions [29,30,31,32,33]. Out of 3765 metabolic genes present in HMR2.0, 3584 were present in the gene expression dataset. The median absolute deviation (MAD) was computed for all metabolic genes. The top 1000 genes based on the MAD score were selected as a seed list for dimension reduction and clustering. This pre-filtering step eliminates the majority of genes and retains only genes with high MAD scores greater than 0.9. We adopted a Non-Negative Matrix Factorization (NMF)-based consensus clustering approach to identify clusters.

NMF is a low-rank approximation technique that has been applied in cancer research for clustering gene expression to uncover the most representative genes of a cluster and identify the cancer subtypes [34]. NMF generates a compressed representation of a given matrix without losing relevant information. It facilitates the automatic extraction of meaningful sparse features from high-dimensional data, resulting in a straightforward interpretation of its factors, which is lacking in other matrix factorization techniques, such as SVD or PCA. In NMF, a data matrix X of dimension m×n (m represents the features and n represents the samples) containing non-negative values (i.e., $x_{ij}>0$) is written as an approximate product of the non-negative matrices W and H as follows:

X ≈WH

Here, W and H refer to the basis/meta-gene matrix and coefficient/meta-profile matrix of the dimensions m×r and r×n, respectively. r denotes the rank of the factor matrices/number of clusters/components, and its value must be an integer with value $0<r\ll \;min\left(m,n\right)$. The objective of NMF is to factorize the matrix X into two low-rank matrices W and H such that it can introduce sparseness into its factors by solving the following non-linear optimization problem:

$$\begin{array}{c} min\;\\ W\in {\mathbb{R}}^{m\times r},\;H\;\in {\mathbb{R}}^{r\times n}\; \end{array}∥X-WH{∥}_{KL}^{2}\;\mathrm{such}\;\mathrm{that}\;W\geq 0\;and\;H\;\geq 0$$

*KL* represents the Kullback–Leibler divergence, which computes the error between the given matrix X and its approximate factors WH.

The consensus clustering combines the output of multiple runs of NMF-based clustering results to produce stable clusters. In each iteration, it subsamples the data and constructs a consensus matrix consisting of pairwise values corresponding to the fraction of times two samples were clustered together when they were subsampled. The NMF-based consensus clustering was carried out using the NMF R package (version 0.25) [35]. The silhouette width, dispersion, and cophenetic correlation coefficient were utilized to decide the optimal number of clusters. We also experimented with six different versions of NMF methods (brunet, KL, lee, Frobenius, offset, and nsNMF). The performance of all methods was evaluated on the same criteria used for determining the number of clusters.

### 2.3. Differential Gene Expression Analysis

The differential gene expression analysis was performed using the DESeq2 R package (version 1.32.0) to identify metabolic alterations between metabolic subtypes [36]. We also performed differential gene expression analysis between 23 pairs of adjacent normal and tumor samples for a comparative study. The differentially expressed genes (DEGs) were identified based on log_2_ fold change (FC) (|log_2_FC|) > 1 and adjusted *p*-value (adj *p*-value) < 0.05. For the GEO dataset, we identified DEGs using the limma R package (version 3.48.3) with criteria of |log_2_FC| > 0.6 and adj *p*-value < 0.05 [37]. We considered different log_2_FC for RNA-Seq and microarray since differences in expression levels can emerge due to the differences in sampling and quantification methodology (sequencing vs. intensity-based). The univariate Cox regression analysis was performed to determine the DEGs significantly associated with the survival outcomes of EC patients. A *p*-value < 0.05 was considered for identifying prognostic genes.

### 2.4. Reporter Metabolite Analysis

We adopted a metabolic-network-topology-based approach to integrate gene expression data with HMR2.0. The gene expression changes between subtypes were mapped to a bipartite undirected graph comprising metabolites and enzymes as nodes for identifying reporter metabolites. Integration was performed using *p*-value and log_2_FC score, obtained from DESeq2 analysis of metabolic subtypes. The reporter metabolite algorithm utilizes an inverse normal cumulative distribution to convert the *p*-value of an enzyme into the z-score [22]. Then, the method identifies the enzymes around each metabolite and computes the -Z-score of that metabolite using the following equation:

(1) $$Z_{metabolite}=\frac{1}{\sqrt{k}}\overset{k}{\underset{i=1}{\sum }}z_{i}$$

Here, k represents the number of enzymes around each metabolite. A total of 100,000 sets of k enzymes were chosen randomly to correct the Z-score of each metabolite by subtracting the mean ($\mu_{k}$) and dividing by the standard deviation ($\sigma_{k}$) of the aggregated Z-score of all sets. The corrected Z-score was transformed into a *p*-value. We selected reporter metabolites with a minimum of 3 neighboring genes and a *p*-value < 0.05.

### 2.5. Association of Metabolic Subtypes with Clinical Information

The log-rank test was performed to assess the survival differences between metabolic subtypes. The Kaplan–Meier Plot was generated using the R package. The relationship of metabolic subtypes with clinical variables such as clinical stage, histological type, histological grade, and age was determined by Fisher’s exact test. Cramer’s V, which measures the relationship between categorical variables, was also computed to quantify the association of the clinical variables with the subtypes [38]. Its values vary from −1 to +1. A value of +1 dictates a stronger association, 0 represents no association, and −1 represents a strong association in the opposite direction.

### 2.6. Genomic Profile of Metabolic Subtypes

The TCGA somatic mutation data and copy number variation (CNV) datasets were used to assess the unique characteristics of metabolic subtypes at the genome level. The number of somatic mutation and CNV samples was 525 and 534, respectively. The “maftool” R Package (version 2.8.5) was employed to analyze and visualize the mutation profile of metabolic subtypes [39]. The differentially mutated genes between subtypes were identified based on the minimum mutation frequency of 10 using Fisher’s exact test (*p*-value < 0.01). We used the Genomic Identification of Significant Targets in Cancer (GISTIC) 2.0 method to identify the significant recurrent CNVs specific to each subtype [40]. The analysis was performed with an amplification/deletion threshold > |0.1|, a confidence interval of 0.99, and a *q*-value < 0.05. The GISTIC results were also visualized with maftools.

## 3. Results

### 3.1. Stratification of EC Samples into Two Metabolic Subtypes with Distinct Survival

**Figure 2:**
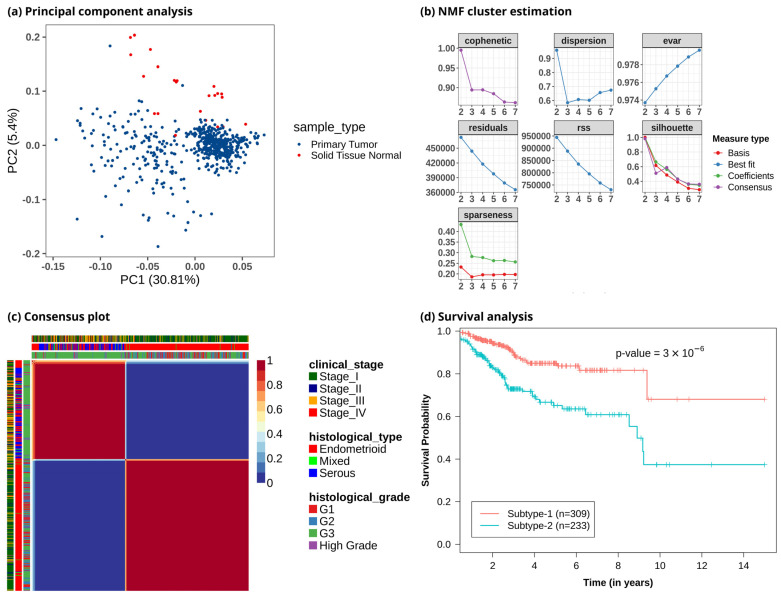
Clustering of EC patients based on the metabolic gene expression profile. (**a**) Principal component analysis reveals metabolic heterogeneity within EC. The *x*-axis represents the first principal component, which captures 30.81% variation, and the *y*-axis represents the second component, which captures 5.4% variation. (**b**) Identifying an optimal number of clusters from different cluster sizes (k = 2–7). The *y*-axis represents the evaluation metric, and the *x*-axis denotes the number of clusters. (**c**) Heatmap of consensus matrix. EC patients are grouped into two distinct clusters. Dark red and blue colors correspond to consensus scores of 1 and 0, respectively. A consensus score of 1 represents samples always occupying the same clusters in all iterations, whereas a consensus score of 0 means that samples do not occur together in any iteration. The plot also includes information on clinical variables corresponding to each patient, represented by color bars on the top and left. (**d**) Kaplan–Meier plot shows metabolic subtypes have significantly different survival probabilities (*p*-value < 0.001).

To study metabolic reprogramming in EC, we analyzed the gene expression data of 3765 metabolic genes in the human genome-scale metabolic model HMR2.0. The principal component analysis (PCA) using metabolic genes showed that normal samples had different metabolic gene expression profiles compared to tumor samples, with most of the variance captured by principal components PC1 and PC2 (Figure 2a and Figure S1). It also indicated that the metabolic profile of EC patients was heterogeneous. To discover the metabolic subtypes within EC, we applied the NMF method to cluster the EC patients using the expression profile of the top 1000 metabolic genes filtered based on the MAD score. We ran different NMF algorithms with default parameters for 2000 iterations. This was repeated 50 times with random subsampling to generate a consensus matrix. Different cluster sizes (2 to 7) were experimented with to tune the optimal number of clusters (Figure 2b). We identified the optimal number of clusters based on silhouette width, dispersion, and cophenetic correlation coefficient. The offset NMF technique achieved the best performance (silhouette width = 0.98, dispersion = 0.96, cophenetic = 0.995) among the different variants of NMF methods, which yielded two clusters (Figure 2c). We also varied the input metabolic genes and observed that considering all or only the top few features decreased the performance (Table S1). However, the optimal number of clusters obtained was always two with almost similar cluster sizes. The 542 EC patients were clustered into two subtypes: metabolic subtype-1 consisted of 309 patients, and metabolic subtype-2 consisted of 233 patients. To assess the survival difference between subtypes, we performed an overall survival analysis. Both subtypes showed significant differences in survival outcomes (*p*-value < 0.001). Metabolic subtype-1 was associated with better survival, while metabolic subtype-2 was associated with poor survival (Figure 2d).

### 3.2. Metabolic Subtypes Show Association with Histological Characteristics

**Figure 3:**
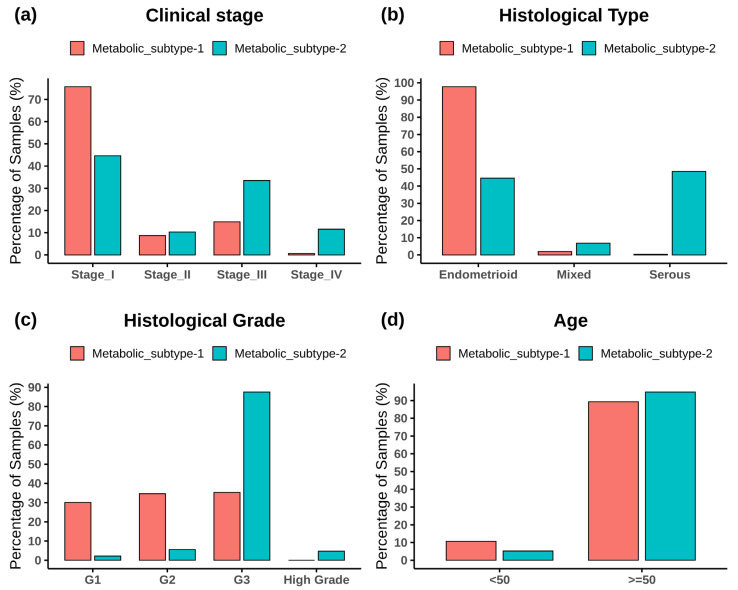
The bar plot shows the distribution of clinical variables in each metabolic subtype. (**a**) Clinical stage, (**b**) histological type, (**c**) histological grade, and (**d**) age.

Figure 3 shows the subtype-wise distribution across stages, histological types, grades, and ages. Fisher’s exact test showed that both subtypes are significantly related to the clinical variables (*p*-value < 0.05) (Table S2). Metabolic subtype-1 is dominated by stage 1 samples, whereas metabolic subtype-2 is dominated by stage 3 and stage 4 samples (Figure 3a). Stage 2 samples are almost equally distributed into both subtypes. The Cramer’s V value of 0.36 shows a moderate association between clinical stage and metabolic subtypes. Metabolic subtype-1 mainly consists of endometrioid samples, while metabolic subtype-2 comprises all histological types (Figure 3b). Both serous and endometrioid types have an almost equal distribution in subtype-2 samples. The Cramer’s V value of 0.61 describes strong relationships between metabolic subtypes and histological types. Metabolic subtype-1 consists primarily of grade 1 to 3 samples (Figure 3c). However, the grade 3 samples are more in metabolic subtype-2 compared to metabolic subtype-1. All high grades samples only belong to metabolic subtype-2. The Cramer’s V value for a histological grade is 0.58, which indicates a strong relationship between them. In addition, we divided the samples into two categories to perform Fisher’s exact test between age and clusters (<50 years and ≥50 years). The association of age with metabolic subtypes is significant (*p*-value = 0.02), but Cramer’s V value for age is very small (0.098), indicating a negligible association of metabolic subtypes with age (Figure 3d).

### 3.3. Metabolic Gene Alterations in EC

**Figure 4:**
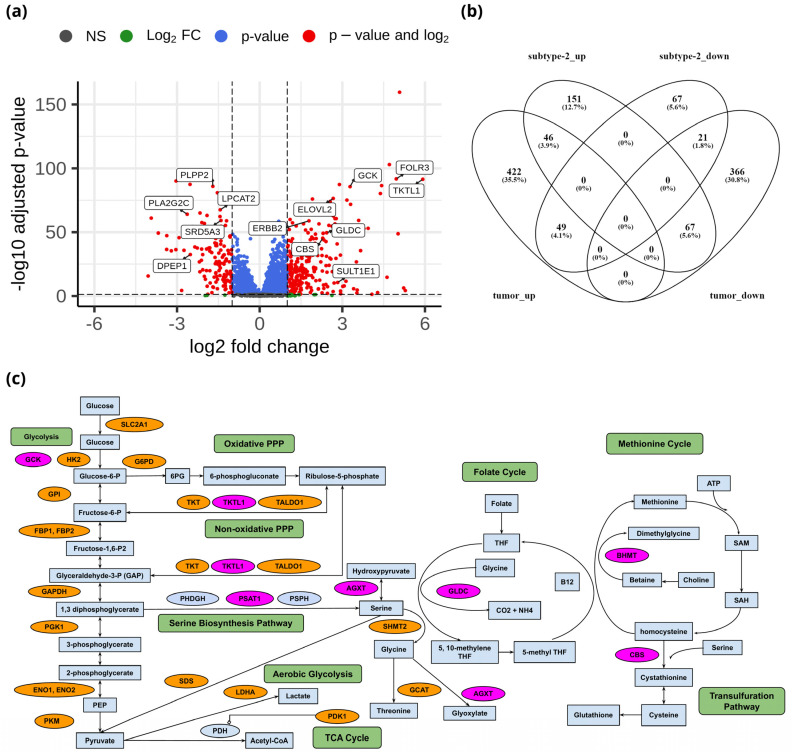
(**a**) Volcano plot showing DEGs between metabolic subtype-1 and subtype-2 samples. (**b**) Venn diagram showing the overlap of DEGs between metabolic subtype-1 vs. metabolic subtype-2 and normal vs. tumor comparisons (subtype-2_up: upregulated genes in metabolic subtype-2, subtype-2_down: downregulated genes in metabolic subtype-2, tumor_up: upregulated genes in the tumor, tumor_down: downregulated genes in the tumor). Venny 2.1 was used to generate the Venn diagrams. (**c**) Metabolic pathways altered in EC and metabolic subtype-2 samples. Orange and pink colors represent upregulated genes in normal vs. tumor conditions and metabolic subtype-1 vs. metabolic subtype-2 conditions, respectively.

To identify the unique metabolic characteristics of metabolic subtypes at the transcriptome level, we performed differential gene expression analysis of 3584 metabolic genes between metabolic subtype-1 and subtype-2. There were 264 upregulated and 137 downregulated genes between metabolic subtype-1 and subtype-2 samples based on the criteria of |log_2_FC| > 1 and adj *p*-value < 0.05 (Data S1). The top candidate genes are shown in Figure 4a. We also analyzed differential gene expression between adjacent normal and tumor samples to study the overlap with metabolic subtypes. We identified 971 DEGs (517 upregulated and 454 downregulated) between normal and tumor samples (Data S1). The overlap of DEGs between both analyses shows that there are only 46 genes that are upregulated in both metabolic subtype-2 and tumor samples (Figure 4b). Most genes (151) were uniquely upregulated in metabolic subtype-2, indicating a different set of genes responsible for cancer progression in metabolic subtype-2 samples.

Genes of the glycolysis pathway (*SLC2A1*, *HK2*, *GPI*, *FBP1*, *FBP2*, *PFKFB1*, *PFKFB2*, *PFKFB4*, *TPI1*, *GAPDH*, *PGK1*, *ENO1*, *ENO2*, and *PKM*) were upregulated in EC (Figure 4c). *PDK1* inhibits oxidative decarboxylation of pyruvate by *PDH* and is upregulated in EC samples along with *LDHA*, which controls the interconversion of pyruvate and lactate. Further, two critical enzymes of the PPP: *G6PD*, which catalyzes the conversion of glucose-6-phosphate into 6-phosphogluconolactone, and *PGD*, which yields ribulose 5-phosphate by catalyzing the oxidative decarboxylation of 6-phosphogluconate, are upregulated in EC samples. PPP plays a role in the synthesis of nucleotides and in generating coenzymes to maintain redox homeostasis. Further, enzymes of the non-oxidative phase of the PPP (*TKT*, *TALDO1*) are also upregulated in EC samples. Interestingly, *TKTL1*, another critical enzyme that connects the PPP with glycolysis by converting D-xylulose 5-phosphate into D-glyceraldehyde 3-phosphate, is upregulated in metabolic subtype-2 samples (log_2_FC = 5.91).

We observed that the genes (*BHMT*, *PSAT1*, *AGXT*, *DAO*, *GLDC*, and *CBS*) involved in glycine, serine, and threonine metabolism were upregulated in metabolic subtype-2 samples (Figure 4c). *PSAT1* catalyzes the conversion of 3-phosphohydroxypyruvate to phosphoserine, which is further used in the formation of serine. Downregulation of *PSAT1* promotes growth inhibition and induces apoptosis [41]. Cells with high *PSAT1* levels increase the ratio of reduced GSH (glutathione) to oxidized GSSG (Glutathione disulfide) and lower ROS. *GLDC* catalyzes the conversion of glycine to 5,10-methyleneteTHF, which is an essential intermediate in the folate cycle. *CBS*, a vital gene of the transsulfuration pathway, catalyzes the conversion of serine to cystathionine, the precursor of L-cysteine. *BHMT* catalyzes the conversion of homocysteine to methionine and dimethylglycine using choline-derived betaine as a methyl donor. We also observed genes involved in glycine, serine, and threonine metabolism (*GLDC*, *PSAT1*, *SHMT2*, *GCAT*, and *SDS*) to be upregulated in EC samples. However, the expression of *GLDC* and *PSAT1* was very high in metabolic subtype-2 samples.

Estrogen sulfotransferase enzyme *SULT1E1* was upregulated in metabolic subtype-2 samples. *SULT1E1* catalyzes the sulfation of estrogens (estradiol and estrone) and catechol estrogens. *UGT1A1* of the glucuronidation pathway was upregulated in metabolic subtype-2 samples. It encodes the UDP-glucuronosyltransferase enzyme, a major contributor to the glucuronidation activity associated with estrogens and catechol estrogens. Cytochrome P450 gene *CYP1A1* is also upregulated in metabolic subtype-2 samples. *CYP1A1* catalyzes the 2-OH hydroxylation of estrogens. This suggests that metabolic subtype-2 may be less related to estrogen exposure compared to metabolic subtype-1 samples. 17β-hydroxysteroid dehydrogenases *HSD17B7* and *HSD17B2*, involved in the interconversion between estrone and estradiol, were upregulated in EC samples. On the other hand, *CYP1B1*, which catalyzes the 4-OH hydroxylation of estrogens, was downregulated in EC samples. The glutathione S-transferase enzymes (*GSTA1*, *GSTA2*, *GSTA3*, *GSTM1*, *GSTM2*, *GSTM3*, and *GSTM5*) were also downregulated in EC samples. GSTs play a role in the inactivation of catechol estrogen. These observations indicate that estrogen exposure plays a critical role in the development of EC.

On the other hand, we observed genes (*GGT1*, *GGCT*, *OPLAH*, *ANPEP*, *G6PD*, *PGD*, *IDH1*, *IDH2*, *GPX2*, *GPX7*, and *GSR*) that control the intracellular content of glutathione through de novo synthesis and regeneration of GSH from GSSG to be upregulated in EC samples, consistent with previous studies [42]. *GGT1*, *GGCT*, *OPLAH*, and *ANPEP* play critical roles in synthesizing glutathione from glutamate, cysteine, and glycine. Similarly, the upregulation of *G6PD*, *PGD*, *IDH1*, and *IDH2* can increase NADPH, which is required to reduce glutathione. *GPX2* and *GPX7* are involved in converting GSH to GSSG to reduce hydrogen peroxide and superoxide radicals, while GSR catalyzes the reduction of GSSG to GSH.

The expression of genes in the urea cycle (*ASS1*, *CPS1*, *OTC*, *SLC25A13*, and *SLC25A15*) is altered in EC samples. Urea cycle dysregulation promotes nitrogen utilization for pyrimidine synthesis [43]. Downregulation of *ASS1* expression promotes cancer proliferation by diversion of its aspartate substrate toward the pyrimidine synthesis pathway [44,45]. *SLC25A13*, which exports aspartate from the mitochondria, is upregulated in EC samples.

### 3.4. Prognostic Metabolic Genes of EC

We performed a univariate Cox regression analysis to discover the prognostic genes, which are differentially expressed between metabolic subtypes. We observed that 225 DEGs in metabolic subtype-2 samples are associated with the survival of EC patients (Data S1). Among them, the high expression of 156 genes is associated with poor survival (hazard ratio > 1), while the high expression of 69 genes is related to better survival outcomes (hazard ratio < 1). There are 86 genes that are only upregulated in metabolic subtype-2 samples and have a significant association with survival outcomes. These include genes of amino acid metabolism (*PNMT*, *DDC*, *BHMT*, *CBS*, *TH*, and *GLS*), estrogen metabolism (*SULT1E1, UGT1A1, CYP1A2,* and *ERBB2*), fatty acid synthesis (*ELOVL2*, *ELOVL4*, and *ELOVL7*), and amino acid transporters (*SLC38A1* and *SLC38A3*). High expression of these genes corresponds to poor survival of EC patients. On the other hand, genes involved in glycerophospholipid metabolism (*LPCAT2*, *PLA2G2C*, *PLA2G2D*, *PLA2G10*, *GPCPD1*, and *PLPP2*) were downregulated in metabolic subtype-2 samples, and their low expression is correlated with poor survival outcomes. We also identified *GCK*, *PSAT1*, *GLDC*, and *UGT3A1* as prognostic markers for EC. They were DEGs in normal vs. tumor and metabolic subtype-1 vs. metabolic subtype-2 comparisons.

### 3.5. Reporter Metabolites

**Figure 5:**
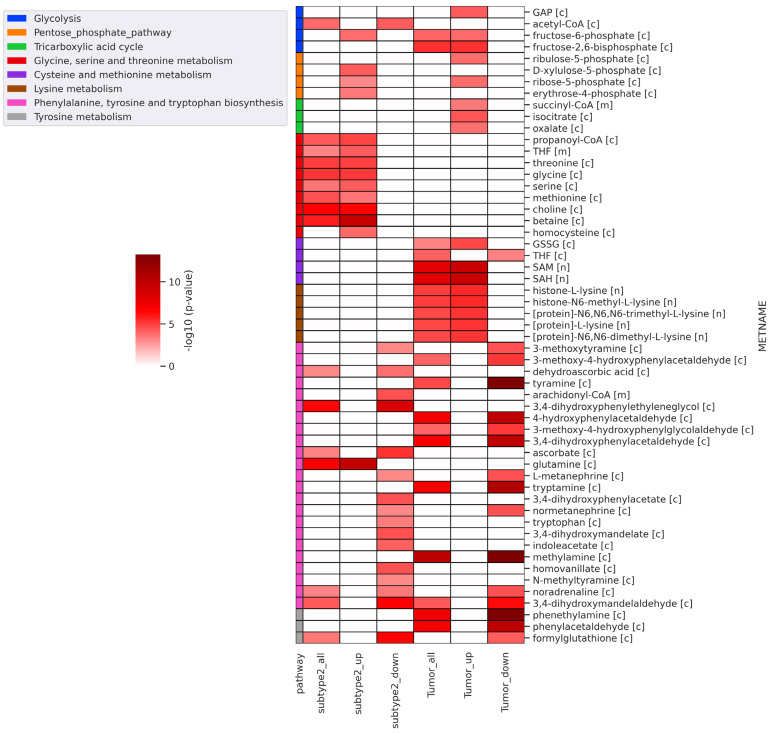
Heatmap of significant reporter metabolites involved in various metabolic pathways obtained for different conditions (subtype2_all: all DEGs of metabolic subtype-1 vs. metabolic subtype-2 condition; subtype2_up: upregulated genes in metabolic subtype-1 vs. metabolic subtype-2 condition; subtype2_down: downregulated genes in metabolic subtype-1 vs. metabolic subtype-2 condition; tumor_all: all DEGs of normal vs. tumor conditions; tumor_up: upregulated genes in normal vs. tumor conditions; tumor_down: downregulated genes in normal vs. tumor conditions). The metabolite name (METNAME) also includes the compartment information: [c]—cytosol, [m]—mitochondria, [n]—nucleus.

Further, we performed reporter metabolite analysis to identify altered metabolites between metabolic subtype-1 and subtype-2 and adjacent normal and tumor samples. The analysis was performed with all DEGs, upregulated DEGs, and downregulated DEGs in metabolic subtype-2 and all tumor samples. We mapped the reporter metabolites with HMR2.0 metabolic pathways to infer the altered pathway. The metabolites of one-carbon metabolism (betaine, Choline, Glycine, serine, threonine, homocysteine, methionine, and THF) and the PPP (fructose-6-phosphate, ribose-5-phosphate, D-Xylulose-5-phosphate, and erythrose-4-phosphate) are reporter metabolites of metabolic subtype-2 samples (Figure 5). Both one-carbon metabolism and PPP are at the crossroads of anabolism and redox homeostasis. We also found glutamine to be a reporter metabolite in metabolic subtype-2 samples. In addition, the metabolites of fatty acid synthesis, fatty acid elongation, omega-3 fatty acid metabolism, and omega-6 fatty acid metabolism are associated with upregulated genes of metabolic subtype-2 samples (Figure S2). In EC samples, we observed SAM and SAH to be reporter metabolites, along with metabolites of glycolysis (fructose-6-phosphate, fructose-2,6-bisphosphate, and Glyceraldehyde-3-phosphate (GAP)), PPP (ribose-5-phosphate and ribulose-5-phosphate), TCA cycle (succinyl-CoA, isocitrate, and oxalate) and lysine metabolism (L-lysine, N6,N6-dimethyl-L-lysine, N6,N6,N6-trimethyl-L-lysine, Histone-L-lysine, and histone-N6-methyl-L-lysine). On the other hand, the metabolites associated with downregulated genes of metabolic subtype-2 samples map to phenylalanine, tyrosine, and tryptophan biosynthesis, sphingolipid metabolism, and glycerophospholipid metabolism (Figure 5 and Figure S2).

### 3.6. Genomic Alterations of Metabolic Subtypes

**Figure 6:**
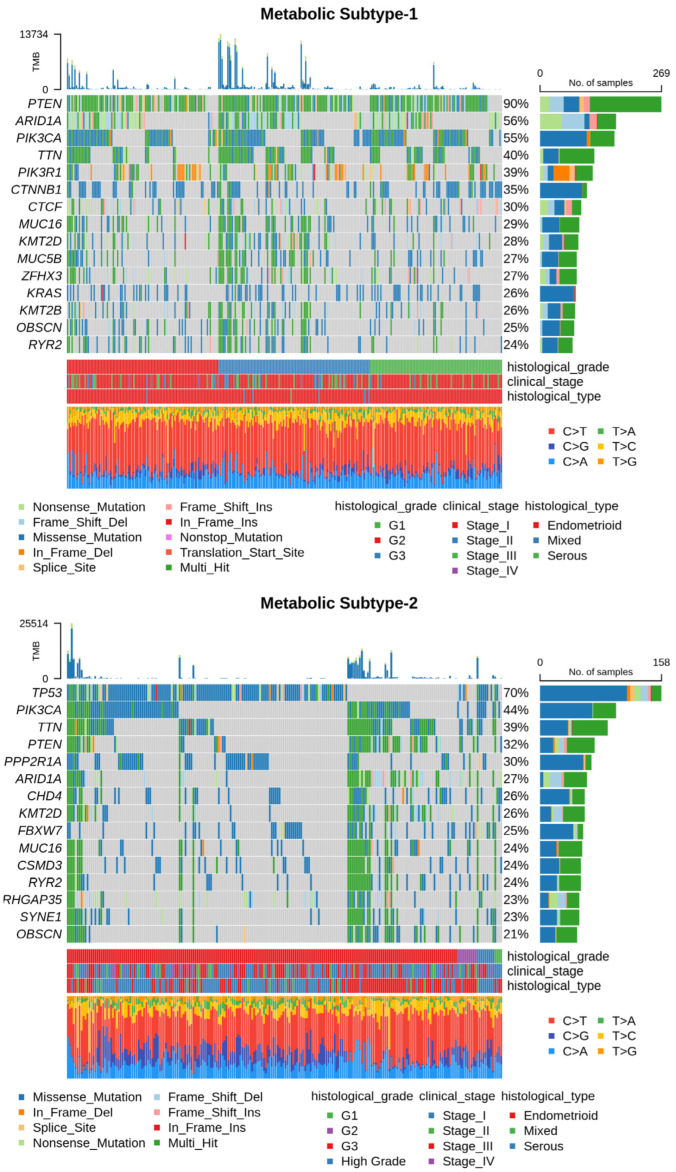
Oncoplot shows frequently mutated genes in metabolic subtype-1 and subtype-2 samples. Clinical subtypes and stages of samples are given along with various mutation types.

Next, we utilized somatic mutation and CNV datasets to characterize each subtype at the genome level. The somatic mutation datasets for metabolic subtype-1 and subtype-2 consisted of 300 samples (out of 309) and 225 samples (out of 233), respectively. Table S3 summarizes the mutation profile of metabolic subtype-1 and subtype-2 samples. The mean Tumor Mutation Burden (TMB) per Mb of metabolic subtype-2 (32.38) was higher compared to metabolic subtype-1 (25.78) samples. The five most frequently mutated genes in metabolic subtype-1 samples were *PTEN* (90%), *AR1D1A* (56%), *PIK3CA* (55%), *TTN* (40%), and *PIK3R1* (39%) (Figure 6). On the other hand, *TP53* (70%), *PIK3CA* (44%), *TTN* (39%), *PTEN* (32%), and *PPP2R1A* (30%) were the top five frequently mutated genes in metabolic subtype-2 samples (Figure 6). *TTN* is frequently mutated in both subtypes.

**Figure 7:**
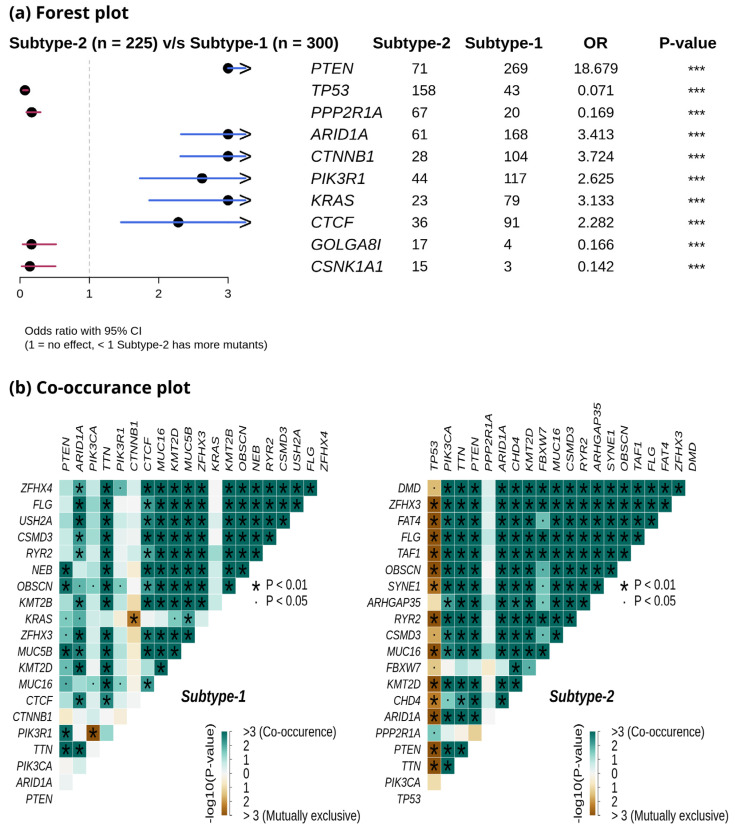
(**a**) Forest plot shows differentially mutated genes between metabolic subtype-1 and subtype-2 samples. The symbol *** represents the *p*-value < 0.001. (**b**) Co-occurrence analysis between frequently mutated genes of metabolic subtype-1 and metabolic subtype-2 samples.

We compared the mutation profiles of metabolic subtypes and identified 72 differentially mutated genes (*p*-value < 0.01) between the subtypes. Out of 72, 16 genes were enriched in metabolic subtype-1, while 56 were differentially mutated in metabolic subtype-2 samples. Interestingly, we found four metabolic genes (*UBIAD1*, *PDK1*, *GOT2*, and *CYP1A1*) to be enriched in metabolic subtype-2 samples. *UBIAD1* plays a crucial role in cholesterol and phospholipid metabolism. It has tumor suppressor function, and its loss leads to tumor progression with elevated cholesterol levels [46]. *GOT2* encodes a mitochondrial enzyme that maintains aspartate-malate shuttle and redox balance and helps cancer cells to proliferate by raising the intracellular aspartate level [47]. *CYP1A1*, a key enzyme of estrogen metabolism, is also associated with breast cancer proliferation and survival [48]. The forest plot shows the differentially mutated genes, including *PTEN*, *TP53*, *PPP2R1A*, *AR1D1A*, *CTNNB1*, *PIK3R1*, *KRAS*, *CTCF*, *GOLGA8*, and *CSNK1A1* (Figure 7a).

**Figure 8:**
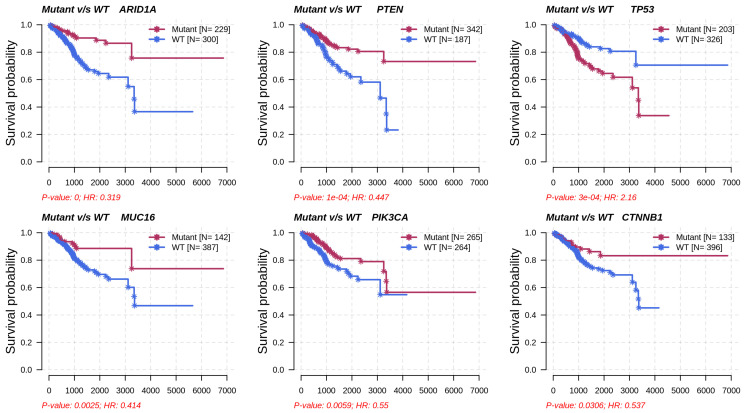
Survival analysis of frequently mutated genes in EC based on their mutation status. HR represents hazard ratio.

Metabolic subtype-2 includes samples with *TP53* mutation, while metabolic subtype-1 has *PTEN* mutations. To further investigate, we analyzed the co-occurrence/mutually exclusive relationships of the 20 most frequently mutated genes in both subtypes. Figure 7b shows that *TP53* and *PTEN* exhibit mutually exclusive behavior (*p*-value < 0.01). *TP53* is also mutually exclusive with other frequently mutated genes in metabolic subtype-2 samples, except for *PPP2R1A*. It has also been reported that *PPP2R1A* mutation is associated with poor survival in breast cancer, lung cancer, and melanoma [49]. The co-occurrence of *PTEN* along with *OBSCN*, *ZFHX3*, *KMT2D*, *MUC16*, and *TTN* was observed in metabolic subtype-1 and metabolic subtype-2 samples (Figure 7b). Further, we performed a survival analysis of the frequently mutated genes to reveal their impact on the survival outcomes of EC patients. Among these genes, six genes had a significant (*p*-value < 0.05) association with survival (Table S4). Patients with *PTEN* mutation have better survival than those without *PTEN* mutation (Figure 8). Similarly, the genes *AR1D1A*, *MUC16*, *PIK3CA*, and *CTNNB1* are related to better survival (hazard ratio < 1) of EC patients (Figure 8). In contrast, patients with *TP53* are significantly associated with poor survival.

**Figure 9:**
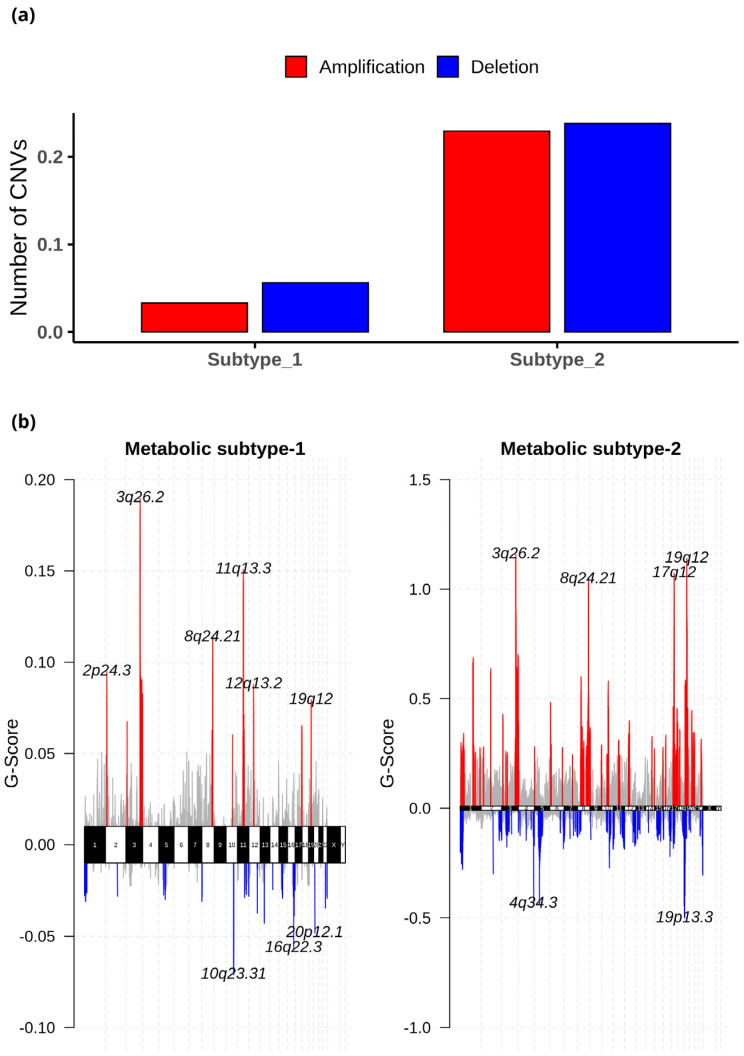
(**a**) The average number of significant CNVs in metabolic subtype-1 and subtype-2 samples. (**b**) The genome plot shows the G-score of significant CNVs of metabolic subtype-1 and metabolic subtype-2 samples for all chromosomes. The red and blue colors represent the amplifications and deletions, respectively. The grey color represents the non-significant CNVs.

The CNV datasets consist of 534 samples, with 303 samples in metabolic subtype-1 and 231 in metabolic subtype-2. We employed GISTIC 2.0 to examine the CNVs in each subtype and identified 27 (10 amplified and 17 deleted) and 108 (53 amplified and 55 deleted) significant CNVs in metabolic subtype-1 and metabolic subtype-2 samples, respectively. These CNVs of metabolic subtype-1 and metabolic subtype-2 mapped to 23 and 103 chromosome loci, respectively. There were 19 common chromosome loci in metabolic subtype-1 and subtype-2 samples. We observed that metabolic subtype-1 samples have very few CNVs (amplifications and deletions) compared to metabolic subtype-2 samples (Figure 9a). The genome plot shows the chromosome locus of the top 10 most significant CNVs (sorted based on *q*-value) of metabolic subtype-1 and subtype-2 samples (Figure 9b). We found four chromosome locations were only altered in metabolic subtype-2 samples: 19p13.3, 17q12, 5q12.1, and 4q34.3. Two CNVs corresponding to chromosome locus 19p13.3 were deleted in over 50% of metabolic subtype-2 samples. Frequent deletion of 19p13 has been observed in ovarian cancer and metastatic melanoma [50,51].

Further, the significant CNVs of metabolic subtype-1 and subtype-2 samples mapped to 1567 and 4264 genes, respectively. Interestingly, we found 11 metabolic gene alterations that also showed consistent changes at the transcriptomic level in metabolic subtype-2 samples. These include eight amplified genes (*PNMT*, *ERBB2*, *ZDHHC19*, *OXCT2*, *CARNS1*, *SLC38A3*, *SLC6A19*, and *SLC6A3*) and three deleted genes (*DPEP1*, *GCNT3*, and *FTMT*) that are upregulated and downregulated in metabolic subtype-2 samples, respectively. *PNMT*, a key enzyme of catecholamine (CAT) synthesis, catalyzes the synthesis of adrenaline from noradrenaline by transferring the methyl group from SAM. We also found *PNMT* to be a prognostic marker for EC. *PNMT*, along with the *ERBB2* gene, is located on chromosome 17q12, which is amplified in 35% of metabolic subtype-2 samples. *ERBB2* controls the activation of estrogen receptors (*ESR1* and *ESR2*) and regulates estrogen metabolism. *ZDHHC19* is amplified in more than 60% of metabolic subtype-2 samples. It is an oncogene involved in leukotriene metabolism and is present on chromosome locus 3q29 along with *PPP1R2*. *OXCT2* plays an essential function in ketone body catabolism by converting fatty acids to ketone bodies. CARNS1(11q13.2), *SLC38A3* (3p21.31), and *SLC6A19* (5p15.33) are involved in the synthesis of carnosine, transportation of glutamine, and neutral amino acid, respectively. The high expression of dopamine transporter *SLC6A3* (5p15.33) has been observed in multiple cancers [52,53]. *DPEP1* (16q24.3) is deleted in more than 60% of metabolic subtype-2 patients, and it catalyzes the conversion of leukotriene D4 to leukotriene E4. *FTMT* (5q12.3) regulates the iron levels in mitochondria and cytosol. The high expression of this gene affects cellular iron homeostasis and inhibits the proliferation of cancer cells [54]. *GCNT3* (15q14) was also deleted in metabolic subtype-2 samples (>50%). It plays an essential role in mucin biosynthesis and is a prognostic marker in EC.

### 3.7. Validation of Metabolic Subtypes

**Figure 10:**
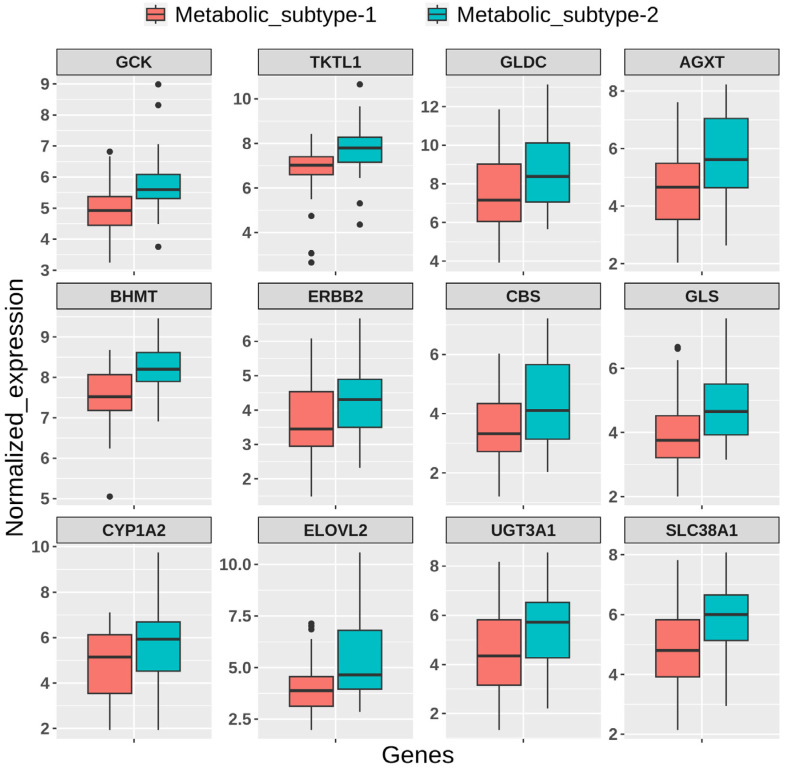
Boxplot showing the expression pattern of candidate genes between metabolic subtypes identified in the GEO validation dataset.

We used independent GEO microarray data to validate our results. Out of 1000 MAD genes used for clustering, 932 metabolic genes that overlap with the GEO dataset were considered for NMF clustering with identical parameters. We obtained two metabolic subtypes with cophenetic and silhouette coefficients of 0.98 and 0.91, respectively, which displayed consistency with the TCGA cohort (Figure S3a). Metabolic subtypes correlated significantly (*p*-value < 0.05) with histological types and grades. Further, the DEGs identified between subtypes overlapped with candidates from the TCGA cohort and showed similar expression patterns (Figure S3b). The expression patterns of some candidate genes are shown in Figure 10.

## 4. Discussion

Previous studies demonstrated that cancer tissues exploit different metabolic pathways to produce energy and biomolecules, along with maintaining redox homeostasis [10,11]. The utilization of various mechanisms depends on intrinsic and extrinsic factors which contribute to heterogeneity in the metabolic profiles of cancer patients. Earlier studies on EC analyzed the difference in the expression of metabolic genes between adjacent normal and tumor samples [15,16]. In this study, we aimed to explore the metabolic heterogeneity within EC. To this end, we applied an unsupervised technique to stratify EC samples into metabolic subtypes based on a genome-scale metabolic model (HMR2.0) and characterized each subtype for survival outcomes, clinical features, metabolic pathways, and genomic alterations. We also compared the metabolic dysregulation of subtypes with changes between normal and tumor conditions. In addition, we leveraged the transcriptomic data to extract reporter metabolites for distinguishing subtypes and normal and tumor conditions.

The consensus clustering of metabolic genes of HMR2.0 based on transcriptomic data of EC revealed two metabolic subtypes (Figure 2). These subtypes significantly correlated to clinical characteristics, including survival, tumor stages, grades, and histological types (Figure 3). This indicates that transcriptomic changes of metabolic genes capture the genotypic-phenotypic relationship. Metabolic subtyping provides molecular insights into the previously established classification scheme of EC. Our analysis of differences between subtypes showed the co-activation of the PPP and one-carbon metabolism in metabolic subtype-2 with poor survival characteristics (Figure 4 and Figure 5). This intersection of metabolic pathways may facilitate the balance between the production of nucleotides and antioxidant species, which support a high proliferation rate and provide defense against oxidative stress [55]. Accordingly, we also observed glycolytic genes to be upregulated in EC, which is consistent with previous studies [15,56]. The candidate genes of PPP include *TKTL1*, which is upregulated in metabolic subtype-2 samples. The transketolase enzyme is part of the non-oxidative branch of PPP that plays a crucial role in the ribose synthesis required for nucleotides. *TKTL1* protein has also been shown to be overexpressed in EC [57]. It is related to poor survival in different cancers, and inhibition of *TKTL1* leads to a significant reduction in the proliferation of many cancers [58]. *GCK*, a prognostic candidate gene of the hexokinase family, is upregulated in metabolic subtype-2 samples, and its high expression is associated with poor survival.

We observed metabolites of one-carbon metabolism as reporter metabolites (Figure 5). Overexpression of genes involved in the serine biosynthesis pathway is also related to poor survival in breast cancer [55]. Serine and glycine provide one-carbon units for nucleotide synthesis through the folate cycle and contribute toward mitochondrial NADPH production. We found folate receptor *FOLR3* to be upregulated in metabolic subtype-2 samples, and its overexpression has been reported in ovarian cancer (Figure 4a) [59]. One-carbon metabolism also contributes to the production of SAM, which is a reporter metabolite in EC samples. SAM is used in the methylation of biomolecules through the methionine cycle. In metabolic subtype-2 samples, we observed choline and betaine as reporter metabolites used in the production of methionine from homocysteine (Figure 5). These are unreported changes in EC, and the upregulation of *BHMT* is associated with poor survival. Serine can also be used in the production of cysteine (through the transsulfuration pathway), which is utilized in the synthesis of glutathione. *CBS*, involved in the first step of the transsulfuration pathway, is upregulated in metabolic subtype-2 samples and is associated with poor survival. The elevated expression of *CBS* is responsible for inducing tumor growth in other gynecological cancers, including ovarian and breast cancer [60]. The changes in one-carbon metabolism may mirror the DNA methylation frequency observed in high-grade tumors [61]. The serum metabolic signature of EC includes serine and homocysteine [62,63]. The upregulation of choline in one-carbon metabolism is also consistent with the metabolic profiling using NMR [17].

The prognostic candidates include genes of estrogen metabolism. EC is characterized by elevated estrogen exposure. Upregulation of *SULT1E1* can inactivate the estrogens in metabolic subtype-2 samples and contribute to the difference in estrogen levels of both subtypes. In metabolic subtype-2 samples, we observed high expression of *ERBB2*, which is correlated with poor survival of EC patients [64]. *GLS*, an essential gene involved in the hydrolysis of glutamine, is also upregulated in metabolic subtype-2 samples. It has been shown that *GLS* in EC is regulated by estrogen [65].

Although only metabolic gene expression data was used for clustering, subtypes identified are described by distinct mutation and CNV patterns capturing the relationship between genomic alterations and metabolic gene expression (Figure 6 and Figure 7). Metabolic subtype-1 samples are associated with *PTEN* mutation and low CNVs, whereas metabolic subtype-2 samples are associated with *TP53* mutation and high CNVs. Both *PTEN* and *TP53* are tumor suppressor genes, and their suppressor functions are associated with cytoplasm and nucleus, respectively [66]. *PTEN* and *TP53* also regulate various metabolic processes to maintain cellular homeostasis [67,68]. We observed a mutually exclusive *PTEN* and *TP53* mutation pattern in metabolic subtype-2 samples. Kurose et al. (2002) showed that *PTEN* and *TP53* mutations are mutually exclusive in breast cancer [69]. We also observed that *PTEN* and *TP53* mutations have opposite survival outcomes in EC (Figure 8). Somatic mutation in *PTEN* is associated with better survival, while *TP53* mutation is related to poor survival. This finding is consistent with the observations made by Risinger et al. (1998) [70]. *ARID1A* mutation exists in the preneoplastic stages, and its loss may not be enough to develop EC [71]. In addition, *PNMT* and *ERBB2* were upregulated, amplified, and present in the same chromosome locus, 17q12, in metabolic subtype-2 samples (Figure 9b). We identified *PNMT* and *ERBB2* as prognostic genes for EC. Gene amplification and coexpression of *PNMT* and *ERBB2* have been observed in breast cancer patients [72]. Further, 17q12 amplification is related to high-grade breast cancer and has been associated with poor outcomes [73].

In the TCGA study, Levine et al. (2013) identified four clusters using CNV data from 363 EC patients [7]. The TCGA cluster-1 samples have a few genomic alterations, while cluster-2 and cluster-3 samples are distinguished by 1q amplification. Cluster-4 comprises many amplified chromosome locations, including 8q11.23 (*SOX17*), 19q12 (*CCNE1*), and 4p16.3 (*FGFR3*) and deletion of *LRP1B*. In our study, we observed very few significant CNVs in metabolic subtype-1 samples and amplification of 1q, 8q11.23 (*SOX17*), 19q12 (*CCNE1*), and 4p16.3 (*FGFR3*) in metabolic subtype-2 samples. These observations indicate that metabolic subtype-1 has the characteristics of TCGA cluster-1 and cluster-2 samples based on CNV data, while metabolic subtype-2 has the characteristics of TCGA cluster-3 and cluster-4 samples. Additionally, metabolic subtype-1 samples exhibit characteristic features of Type 1 EC, while metabolic subtype-2 samples have the characteristics of Type 2 EC based on the association of clusters with survival outcomes, mutation status, and distinct CNVs.

## 5. Conclusions

Our study revealed the metabolic heterogeneity within EC and identified subtypes with distinct transcriptomic, genomic, and clinical features. Although there are fewer mutations, amplifications, and deletions in metabolic genes, the metabolic changes observed at the transcriptome level are associated with the overall genomic changes observed in EC, highlighting the relationship between different data. Metabolic subtyping also suggested that some early and late-stage samples are clustered together, which requires further investigation to understand the molecular variation. Metabolomic profiling of EC tumor samples is required to validate reporter metabolites. The study can be further extended to map the flux level changes using constraint-based modeling and to other versions of human GEMs. Recon3D is another GEM used to investigate metabolic changes in disease conditions [74,75]. Recently, Human-GEM 1.0.2 has been proposed by combining GEMs, including HMR2.0 and Recon3D [76]. A comprehensive elucidation of dysregulated metabolic pathways, which correlate with clinical outcomes, will assist in diagnosing and treating EC.

## Data Availability

The datasets analyzed in the current study are available from public repositories: https://portal.gdc.cancer.gov/ (accessed on 11 February 2022) and https://www.ncbi.nlm.nih.gov/geo/ (accessed on 11 February 2022). The source codes are provided in the GitHub repository: https://github.com/Akankxha/EC_metabolic_subtype_analysis.

## Supplementary Materials

The following supporting information can be downloaded at: https://www.mdpi.com/article/10.3390/metabo13030409/s1, Figure S1: Principal component analysis results; Figure S2: Reporter metabolites involved in lipid metabolism; Figure S3: Validation of metabolic subtypes in an independent GEO dataset; Table S1: NMF results with different number of input genes; Table S2: Fisher exact test and Cramer’s V results; Table S3: Summary of average mutation types; Table S4: Survival analysis of frequently mutated genes; Data S1: List of differentially expressed and prognostic gene.

## Abbreviations

EC	Endometrial Cancer
TCGA	The Cancer Genome Atlas
GEO	Gene Expression Omnibus
HMR2.0	Human genome-scale metabolic model version 2.0
MAD	Median Absolute Deviation
NMF	Non-negative Matrix Factorization
PCA	Principal Component Analysis
DEG	Differentially Expressed Gene
FC	Fold Change
GSH	Glutathione
GSSG	Glutathione disulfide
TMB	Tumor Mutation Burden
CNV	Copy Number Variation
GISTIC	Genomic Identification of Significant Targets in Cancer
PPP	Pentose Phosphate Pathway
TCA	Tricarboxylic Acid
