# Supplementary material for: Identification and Characterization of Metabolic Subtypes of Endometrial Cancer Using a Systems-Level Approach
Source: Metabolites. 2023 Mar 9;13(3):409. doi: 10.3390/metabo13030409 (PMC10054278; doi:10.3390/metabo13030409)
Supplement: Supplementary file 1 [file metabolites-13-00409-s001.zip › Supplementary_files/Supplementary_Information.pdf]

Supplementary Information

Identification and Characterization of Metabolic Subtypes of Endometrial Cancer using a Systems-Level Approach

Akansha Srivastava and P K Vinod*

Centre for Computational Natural Sciences and Bioinformatics, IIIT, Hyderabad-500032, India

Supplementary Tables

Table S1: NMF results with different number of top input genes.

Input genes	silhouette_coef	cophenetic	dispersion	cluster1_size	cluster2_size
100	0.9788	0.9936	0.9544	315	227
1000	0.9833	0.9956	0.9613	309	233
2000	0.9734	0.9942	0.9399	314	228
3584	0.9388	0.9883	0.8408	303	239

Table S2: Fisher exact test and Cramer's V to determine and quantify the association of subtypes with clinical variables.

Clinical Variables	p-value	Cramer's V
Clinical Stage	4.117 e-10	0.361
Histological Types	2.2 e-16	0.614
Histological Grade	2.2 e-16	0.585
Age (>=50)	0.0269	0.098

Table S3: Summary of average mutation types in subtype-1 and subtype-2 samples

Mutation types	Subtype-1	Subtype-2
Frame_Shift_Del	38.42	26.018
Frame_Shift_Ins	14.997	10.72
In_Frame_Del	3.287	2.396
In_Frame_Ins	0.207	0.191
Missense_Mutation	769.017	1006.698
Nonsense_Mutation	77.157	86.222
Nonstop_Mutation	0.767	0.844
Splice_Site	18.52	25.431
Translation_Start_Site	0.7	0.907
TMB	923.07	1159.427
TMB per mb	25.78	32.38

Table S4: Survival analysis based on the mutation status of frequently mutated genes in EC. Genes with p-value < 0.05 have a significant association with survival.

Genes	p-value	Hazard's Ratio (HR)	Wild Type (WT)	Mutant
ARID1A	3.77E-06	0.319	300	229
PTEN	0.000131	0.447	187	342
TP53	0.000303	2.16	326	203
MUC16	0.0025	0.414	387	142
PIK3CA	0.00591	0.55	264	265
CTNNB1	0.0306	0.537	396	133
ZFHX3	0.0636	0.6	402	127
KMT2D	0.0672	0.617	385	144
TTN	0.116	0.697	319	210
PIK3R1	0.975	1.01	367	162

Supplementary Figures

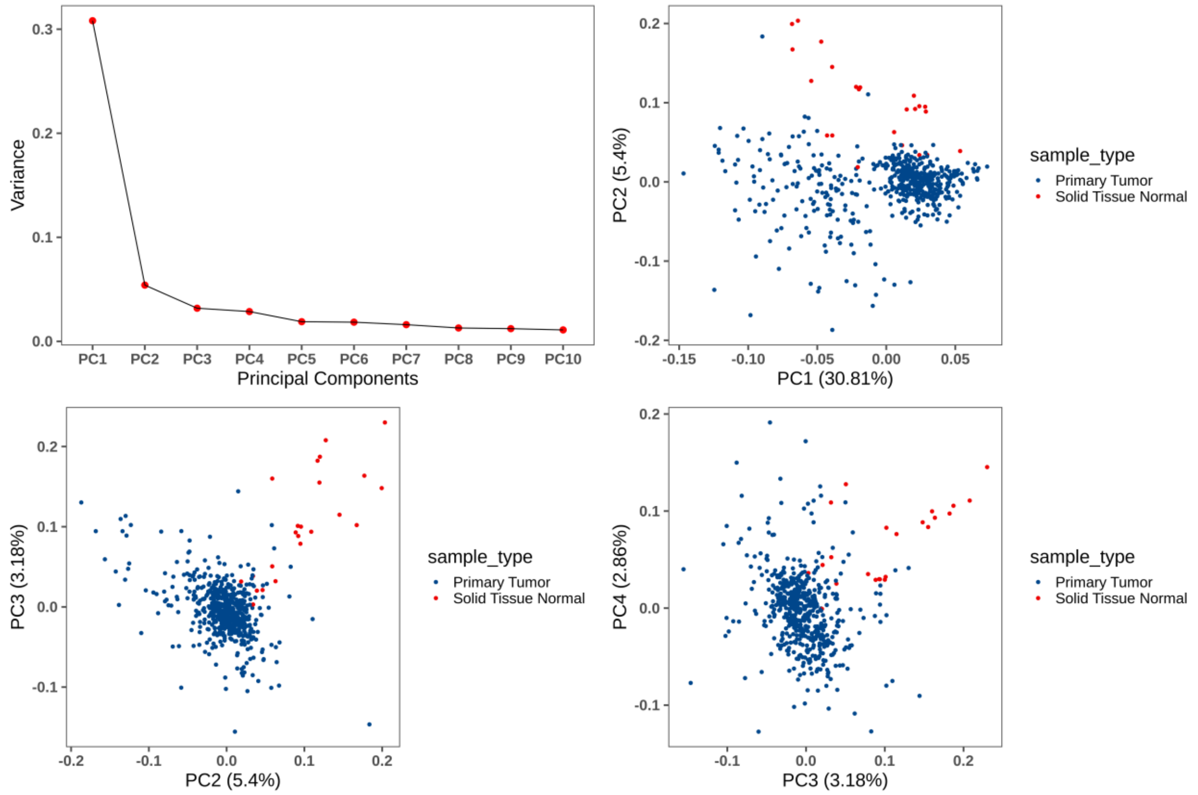

Figure S1: Principal component analysis (PCA) for the metabolic profile of EC patients. The Scree plot shows a sharp drop in variance after the first principal component. Subsequent PCA plots show the distribution of EC patients for different pairs of principal components, revealing that normal samples have different metabolic gene expression profiles compared to tumor samples.

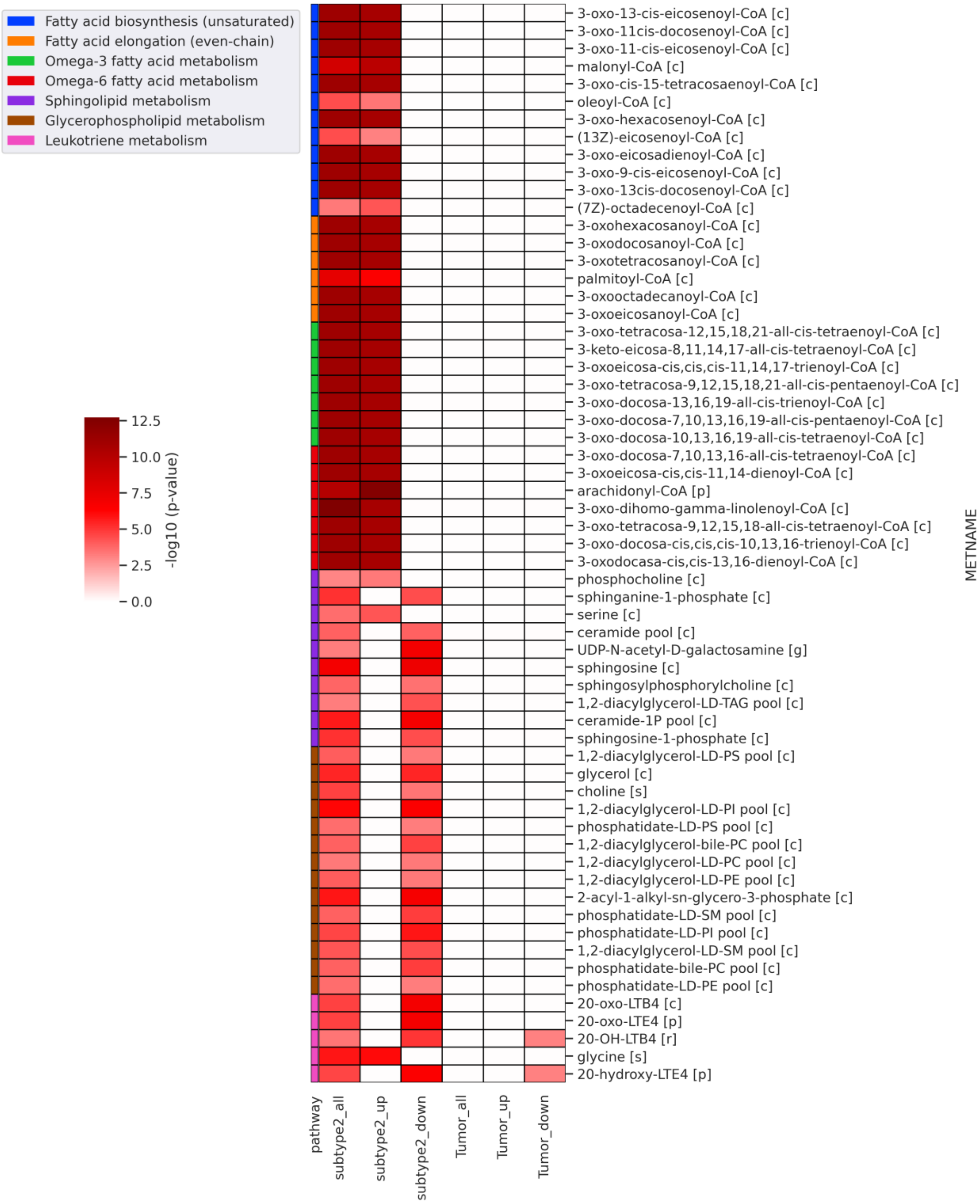

Figure S2: Heatmap of significant reporter metabolites in lipid metabolism obtained for different conditions (subtype2_all: all DEGs of subtype-1 vs. subtype-2 condition, subtype2_up: upregulated genes in subtype-1 vs. subtype-2 condition, subtype2_down: downregulated genes in subtype-1 vs. subtype-2 condition, tumor_all - all DEGs of normal vs. tumor conditions, tumor_up: upregulated genes in normal vs. tumor conditions, tumor_down: downregulated genes in subtype-1 vs. subtype-2 conditions). The metabolite name (METNAME) also includes the compartment information, [c] - cytosol, [m] - mitochondria, [n] - nucleus, [p] - peroxisome, [g] - Golgi apparatus, [s] - extracellular, [r] - endoplasmic reticulum.

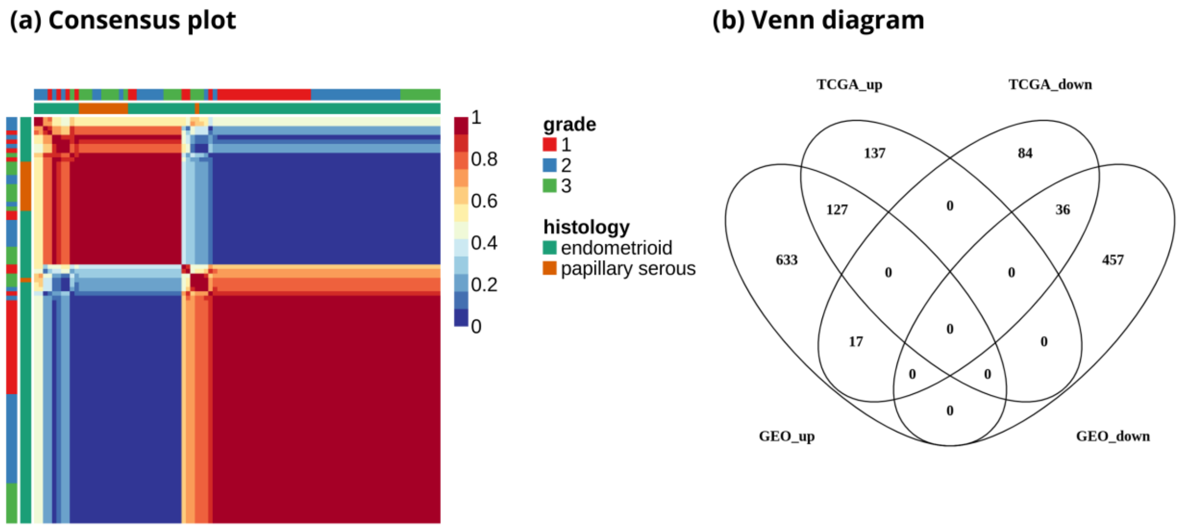

Figure S3: Validation of metabolic subtypes in an independent GEO dataset. (a) The consensus plot shows that tumor samples are clustered into two groups. (b) Comparison of DEGs in TCGA and GEO datasets of EC (TCGA_up: upregulated genes between subtypes in TCGA cohort, TCGA_down: downregulated genes between subtypes in TCGA cohort, GEO_up: upregulated genes between subtypes in GEO dataset, GEO_down: downregulated genes between subtypes in GEO dataset).
